# DSIP-Like KND Peptide Reduces Brain Infarction in C57Bl/6 and Reduces Myocardial Infarction in SD Rats When Administered during Reperfusion

**DOI:** 10.3390/biomedicines9040407

**Published:** 2021-04-09

**Authors:** Elena A. Tukhovskaya, Elvira R. Shaykhutdinova, Alina M. Ismailova, Gulsara A. Slashcheva, Igor A. Prudchenko, Inessa I. Mikhaleva, Oksana N. Khokhlova, Arkady N. Murashev, Vadim T. Ivanov

**Affiliations:** 1 Biological Testing Laboratory, Branch of Shemyakin and Ovchinnikov Institute of Bioorganic Chemistry, Russian Academy of Sciences, Pushchino, Prospekt Nauki, 6, 142290 Moscow, Russia; shaykhutdinova@bibch.ru (E.R.S.); ismailowa.a.m@yandex.ru (A.M.I.); slashcheva_ga@mail.ru (G.A.S.); khohlova@bibch.ru (O.N.K.); murashev@bibch.ru (A.N.M.); 2 Laboratory of Peptide Chemistry, Shemyakin and Ovchinnikov Institute of Bioorganic Chemistry, Russian Academy of Sciences, Miklukho-Maklaya Street, 16/10, 117997 Moscow, Russia; iaprud@yandex.ru (I.A.P.); inessamikh@rambler.ru (I.I.M.); ivavt37@gmail.com (V.T.I.)

**Keywords:** Delta-Sleep-Inducing Peptide (DSIP), KND peptide, myocardial infarction, stroke

## Abstract

A structural analogue of the DSIP, peptide KND, previously showed higher detoxification efficacy upon administration of the cytotoxic drug cisplatin, compared to DSIP. DSIP and KND were investigated using the model of acute myocardial infarction in male SD rats and the model of acute focal stroke in C57Bl/6 mice. A significant decrease in the myocardial infarction area was registered in KND-treated animals relative to saline-treated control animals (19.1 ± 7.3% versus 42.1 ± 9.2%). The brain infarction volume was significantly lower in animals intranasally treated with KND compared to the control saline-treated animals (7.4 ± 3.5% versus 12.2 ± 5.6%). Injection of KND in the first minute of reperfusion in the models of myocardial infarction and cerebral stroke reduced infarction of these organs, indicating a pronounced cardioprotective and neuroprotective effect of KND and potentiality for the treatment of ischemia-reperfusion injuries after transient ischemic attacks on the heart and brain, when administered during the reperfusion period. A preliminary pilot study using the model of myocardial infarction with the administration of DSIP during occlusion, and the model of cerebral stroke with the administration of KND during occlusion, resulted in 100% mortality in animals. Thus, in the case of ischemia-reperfusion injuries of the myocardium and the brain, use of these peptides is only possible during reperfusion.

## 1. Introduction

Peptide KND is a structural analogue of DSIP, differing from it by two amino acid residues (WAGGDASGE, DSIP; WKGGNASGE-KN-DSIP or KND), which was synthesized in the Institute of Bioorganic Chemistry [1]. The KND peptide is a 324–332 fragment of the human lysine-specific histone demethylase 3B. This enzyme belongs to the family of JmjC-domain-containing histone demethylases that are present in tissues of different mammals and are encoded by the *JMJD1B* gene [2]. Previous studies have demonstrated that the biological activity of the KND peptide is superior to that of DSIP. Thus, the antioxidant and anticonvulsant activity of the KND peptide, as well as its effect on animal behavior, have been shown to be more pronounced in comparison to DSIP [3]. The KND peptide provided a two-fold reduction in the mortality of animals after the administration of anticancer drug cisplatin at a dose of LD 50 [1]. In Wistar rats, DSIP has been reported to reduce mortality, decrease the electrical activity of the brain, and increase cerebral blood flow in ischemic brain under conditions of global cerebral ischemia aggravated by pre-stress [4]. An even earlier study demonstrated a high potential of DSIP to reduce mortality when administered preventively 1 h before global cerebral ischemia in Wistar rats [5]. Given that KND is a more active analog of DSIP, it is of high interest to study its effect on the outcome of ischemia-reperfusion injuries that develop after transient myocardial infarction and transient focal cerebral stroke.

## 2. Materials and Methods

### 2.1. Animals

Mature male SD rats, 8–9 weeks old and weighing 300–350 g, were used in the myocardial infarction model study. Mature male C57Bl/6 mice, 8–9 weeks old and weighing 30–35 g, were used in the focal stroke model study. All animals were SPF and were obtained from the Pushchino nursery of laboratory animals (Pushchino, Russia). All procedures and manipulations with animals were approved by the Committee for Control over Care and Use of Laboratory Animals of BIBCh RAS and were carried out in accordance with the EU Directive 2010/63/EU for animal experiments and Animal Control and Use Committee (IACUC) protocol number 695/19. The Biological Testing Laboratory-Branch of Shemyakin and Ovchinnikov Institute of Bioorganic Chemistry, Pushchino, Moscow Region, Russia, where experiments were followed, has AAALAC accreditation (https://www.aaalac.org/accreditation-program/directory/directory-of-accredited-organizations-search-result/?nocache=1#home_acc_dir_search, last renewal of accreditation on 17 November 2017).

### 2.2. Myocardial Infarction Model

#### 2.2.1. Experimental Design-Groups and Doses

A total of 21 mature male SD rats were used. The animals were divided into three groups: group 1 was a control (*n* = 5), group 2 received KND intraperitoneally at a dose of 150 μg/kg (*n* = 8), and group 3 received DSIP intraperitoneally at a dose of 150 μg/kg (*n* = 8) (Table 1). The doses of peptides were chosen based on previous studies, in particular, on the study devoted to the detoxification properties of peptides, in which peptides were injected in mice four times at a course dose of 800 μg/kg [1]. We chose an average dose of 150 μg/kg with a mouse-to-rat conversion factor of 2 (average rat dose = average mouse dose/2). We reduced the dose for rats to 150 μg/kg, since, in our study, we planned a single, instead of four-fold, drug injection. All animals were subjected to the procedure of myocardial infarction simulation by transient occlusion of the left coronary artery for 25 min, followed by 2 h of reperfusion. The vehicle or peptides were injected intraperitoneally in the first minute of reperfusion at a dose of 150 μg/kg in a volume of 5 mL/kg. At the end of the reperfusion period, the heart was removed and stained, and sections were prepared for assessment of the extent of injury.

#### 2.2.2. Procedure of Myocardial Infarction Simulation

**Figure 1:**
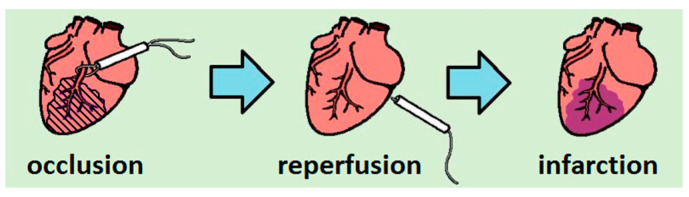
Schematic representation of heart infarction production in a rat’s heart.

Acute myocardial infarction was induced through the left coronary artery (LCA) occlusion with its subsequent reperfusion attended by mechanical ventilation at a respiratory rate of 60–65 breaths per minute and a tidal volume of 10 mL/kg (Rodent Ventilator UGO BASILE 7025). A polyethylene catheter was implanted in the femoral vein for the subsequent injection of dye. Access to the coronary vessel was provided by thoracotomy in the left fourth intercostal space. After opening the chest, the pericardium was incised and the localization of the common trunk of the LCA was determined, under which a thin polyamide thread (ETHILON 6-0) was introduced using an atraumatic needle. The ends of the thread were placed in a PE-10 polyethylene tube, thus forming an occluder to create reversible myocardial ischemia (Figure 1). For occlusion, the threads were tightened and fixed with a hemostatic clamp. The experiments simulated reversible ischemia, followed by reperfusion (restoration of blood flow by releasing the artery). The occlusion lasted 25 min, followed by reperfusion for 120 min. The presence of an infarction was monitored visually by cyanosis of the left ventricle. During the experiment, the body temperature of animals was monitored using a rectal sensor maintained at 37 ± 1 °C with a temperature-controlled surface.

#### 2.2.3. Determination of the Area at Risk, the Infarction Size, the Total Area of the Left Ventricle

In all animals, the total area of the left ventricle, the area at risk, and the infarction size were calculated using the double staining technique with 2% methylene blue and 1% triphenyltetrazolium chloride (TTC) [6,7]. To identify the area at risk (the area of myocardial ischemia upon clamping of the LCA), the coronary artery was ligated in animals at the end of the reperfusion period and a 2% solution of methylene blue, which stains the blood-supplied part of the myocardium blue, was injected into the venous catheter. The heart was then quickly removed, washed in saline, and 2 mm sections were cut in the transverse direction. The obtained sections were incubated for 15 min in a 1% solution of 2,3,5-TTC (pH 7.4), staining the viable myocardium with preserved activity of NAD-dependent enzymes in the area at risk a bright red (brick-red) color at 37 °C, after which they were fixed in 10% formalin for 5 min. Stained sections were placed between glass slides and scanned on a HP LaserJet 3055 scanner. The resulting images were processed using Image TOOL v. 2.0 software (freely available on the Internet). For each section, the area of the left ventricle, the infarction size (unstained with 2,3,5-TTC, located within the area at risk), and areas at risk (stained with 2,3,5-TTC) were calculated. Data are presented as percentage ratios of the size of the infarction area to the total size of the area at risk (IA/AAR, %) [8,9]. The presence of the cardioprotective properties of KND was assessed by a decrease in the IA/AAR ratio [10].

### 2.3. Cerebral Stroke Model

#### 2.3.1. Experimental Design—Groups and Doses

A total of 36 mature male C57Bl/6 mice were used in the cerebral stroke model and animals were divided into three groups: in control group 1 (saline, *n* = 16) half of the mice received saline intranasally with 170 μL/kg—about 5 μL per mouse with an average weight of 30 g (2.5 μL in each nostril), while the other half were injected intraperitoneally with 5 mL/kg; mice in group 2 were injected intraperitoneally with KND at a dose of 300 μg/kg (*n* = 12); and in group 3 mice received KND intranasally at a dose of 300 μg/kg in 170 μL/kg—about 5 μL per mouse with an average weight of 30 g (2.5 μL in each nostril) (*n* = 8) (Table 2). The dosage of KND for mice was chosen based on data from a study that used a rat myocardial infarction model, and we used a rat-to-mouse conversion factor of 2 (mouse dose = rat dose × 2). All animals were subjected to a surgical procedure for simulation of occlusion of the middle cerebral artery, which lasted 2.5 h, followed by reperfusion for 24 h. In the first minute of reperfusion, the animals were treated with either the carrier (saline) or KND intraperitoneally or intranasally according to group specificity. Twenty-four hours after the start of reperfusion, all animals were euthanized by CO_2_ inhalation and we carried out brain sampling for analysis of the volume of cerebral infarction.

Animals that received the saline carrier intraperitoneally and intranasally were combined into one group for calculation, since there were no differences in the volume of cerebral infarction between them. 

#### 2.3.2. Procedure of Acute Focal Stroke Simulation in Mice

**Figure 2:**
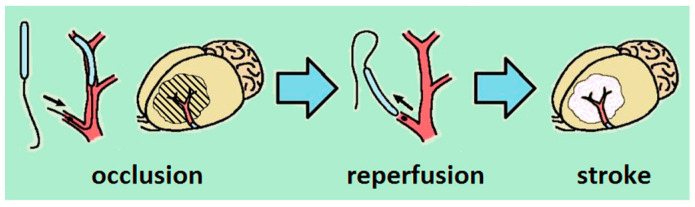
Schematic representation of cerebral stroke production in mouse brain.

Acute focal stroke modeling was performed in male C57Bl/6 mice. General scheme is shown in Figure 2. The animals were anesthetized with a Zoletil 100/Xylazine mixture injected intramuscularly. The hair from the right temple was removed and a small skin incision was made in that area. The temporal muscle was then incised and the open area of the temporal bone was dried with a cotton swab. A flowmeter transducer was attached to the dried bone area to measure local cerebral blood flow (LCBF). A PowerLab 8/35 (ADInstruments, Australia) system for data recording and analysis, and a single-channel laser Doppler blood flowmeter (ADInstruments) and a standard needle probe (ADInstruments) were used. Stroke simulation was performed, as described previously [11,12], with minor modifications. The mouse was fixed in a supine position on an operating table. LCBF was recorded throughout the operation. A skin incision was made in the cervical region, the connective tissues were spread apart, and the common carotid artery (CCA), external carotid artery (ECA), and internal carotid artery (ICA) were isolated from the right side. All three arteries were taken for free ligatures. A region of the ECA was ligated at two points close (about 2 mm) to the bifurcation. The occipital artery was ligated permanently, and the pterygopalatine artery was ligated for the duration of surgery. Monofilaments 702356PK5Re (Doccol, USA) were used for occlusion. The occluder was a silicone monofilament with a diameter of 0.06–0.09 mm and a length of 20 mm, with rubber coating 0.23 ± 0.01 mm in diameter applied over 5–6 mm from one end. This type of occluder is suitable for performing transient occlusion in animals weighing 30 ± 5 g. A small incision was made on the ECA between two ligatures and an occluder was inserted into it. The occluder was passed through the bifurcation into the ICA and gently pushed until there was slight resistance (approximately 10–12 mm). In this case, a blockade of the MCA occurred. The occlusion was considered successful when the relative cerebral blood flow reduced by 70%–80%. After 2.5 h, the occluder was carefully removed through the ECA and the ligatures were tightened on both sides of the opening. Ligatures were removed from the CCA, ICA, and pterygopalatine artery. Restoration of the blood flow to 80–120% of the original level was considered a successful completion of the OMCA procedure. In the first minute of reperfusion, the animals received either KND at a dose of 300 μg/kg or saline (intraperitoneally or intranasally). Following the operation, the animals were placed in a cage with free access to food and water.

#### 2.3.3. Determination of the Volume of Cerebral Infarction

At the end of the reperfusion period (24 h after occlusion removal), the animals were euthanized by CO_2_ inhalation in a special chamber. The brain was removed from the cranium, the olfactory bulbs and cerebellum were separated, and the hemispheres were cut coronally into 5 regions, each approximately 1 mm thick. The obtained sections were incubated in 1% solution of 2,3,5-TTC (pH 7.4) for 5–10 min, inverting the sections in the middle of the staining incubation period. Viable tissue areas were stained in red, while non-viable regions remained unstained. Stained sections were then scanned with a scanner (LaserJet M1132 MFP, HP) and processed using 3D-reconstruction program Reconstruct (freely available on the Internet). The volume of the whole right hemisphere and infarction were calculated separately. The infarction volume was calculated as the percentage of the volume of the infarction compared to the whole of the right hemisphere.

### 2.4. Preliminary Studies with the Introduction of DSIP and KND during the Occlusion Period

Before starting the investigation using the myocardial infarction model, a simulation of myocardial infarction was implemented in 10 animals, and DSIP was injected at different time points during the period of coronary artery occlusion. All animals died within 30 min after administration of DSIP during the period of occlusion. Before initiating the study using the stroke model, focal cerebral stroke was simulated in 4 male C57Bl6 mice. These animals were injected with KND intraperitoneally at a dose of 300 μg/kg 60 min after the onset of occlusion, which lasted 2.5 h followed by reperfusion. All of these animals died within a few hours of the start of the experiment. Upon obtaining the results, it was decided to only carry out the studies with the introduction of DSIP and KND during the reperfusion period. The death of all animals treated with DSIP and KND during the occlusion period in both models (myocardial infarction and cerebral stroke) indicates the danger of using these drugs during acute myocardial and cerebral ischemia.

## 3. Results

### 3.1. Heart Infarction

**Figure 3:**
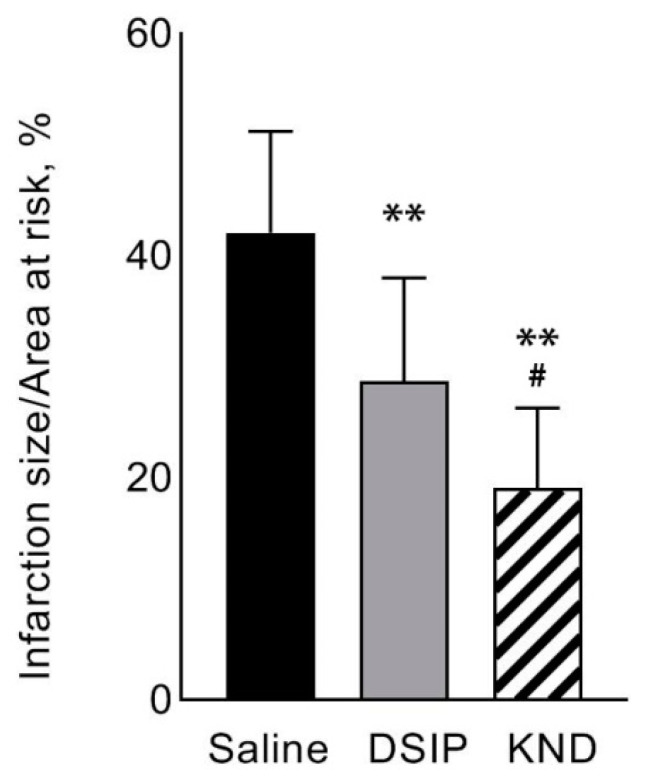
The ratio of the infarction size to the area at risk for three experimental groups: a control group, which received saline (black bar, *n* = 5); a group that received DSIP peptide at a dose of 150 µg/kg/5 mL once in the first minute of reperfusion (gray bar, *n* = 8); and a group that received KND peptide at a dose of 150 µg/Kg/5 mL once in the first minute of reperfusion (striped bar, *n* = 8). ** *p* ≤ 0.01 relative to the control group according to the ANOVA Fisher LSD post-hoc test; # *p* ≤ 0.05 relative to the group that received DSIP, according to an ANOVA Fisher LSD post-hoc test.

The percent ratio of IA/AAR was significantly lower in animals that received DSIP (28.7 ± 9.3% compared to 42.1 ± 9.2% in the control group, *p* = 0.01) and KND (19.1 ± 7.3% in the KND-treated group compared to 42.1 ± 9.2% in the control saline-treated group, *p* = 0.0002) in the first minute of reperfusion. The effect of IA/AAR reduction was significantly greater in the group administered with KND compared to the group treated with DSIP (19.1 ± 7.3% in the KND-treated group vs. 28.7 ± 9.3% in the DSIP-treated group, *p* = 0.04) (Figure 3).

**Figure 4:**
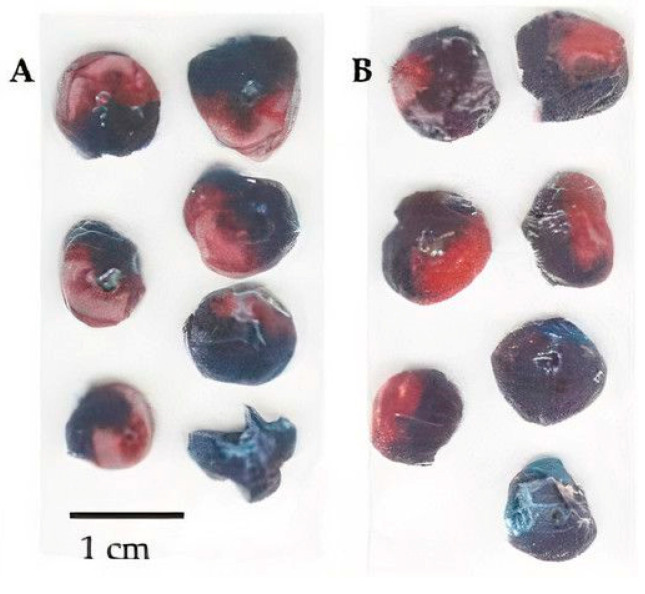
Heart sections double-stained (methylene blue + 1% TTC) for assessment of the area at risk and the infarction size. Dark area—methylene blue-stained blood-supplied part of the myocardium, red area—TTC-stained viable tissue (AAR), white area—unstained non-viable tissue (IA). (**A**) Control animals that received saline; (**B**) animals that received KND peptide once at a dose of 150 µg/kg/5 mL in the first minute of reperfusion.

Figure 4 shows scans of myocardial sections from a control animal and an animal treated with KND.

### 3.2. Stroke

**Figure 5:**
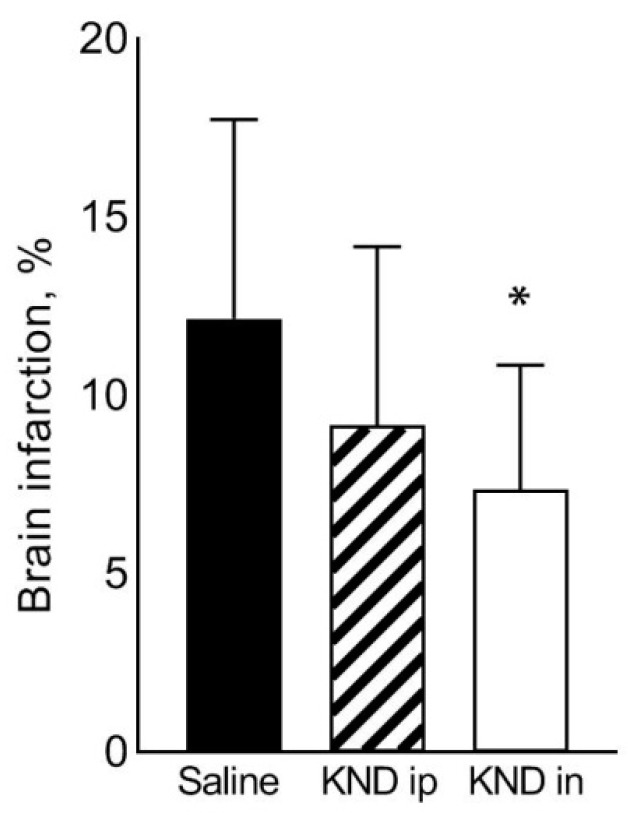
Volume of infarction in the right hemisphere of the brain in C57Bl/6 mice, calculated as a percentage of the volume of damage in the right hemisphere to the total volume of the right hemisphere. The control group (black bar) was subjected to stroke and saline treatment (*n* = 16), the next group (striped bar) was treated with KND peptide intraperitoneally once in the first minute of reperfusion at a dose of 300 μg/kg/5 mL (*n* = 12); and the final group (white bar) received KND peptide intranasally once in the first minute of reperfusion at a dose of 300 μg/kg/5 μL (*n* = 8). * *p* ≤ 0.05 relative to the control group according to a Mann–Whitney U test.

After intraperitoneal administration of KND, the infarction volume in the right hemisphere decreased in comparison to the control animals. However, the differences were not statistically significant (9.2 ± 5.0% in the KND-treated compared to 12.2 ± 5.6% in the saline-treated control group). Upon intranasal administration of KND, the infarction volume in the right hemisphere significantly decreased in comparison to the control animals (7.4 ± 3.5% in the KND-treated group vs 12.2 ± 5.6% in the control saline-treated group, *p* = 0.02) (Figure 5).

**Figure 6:**
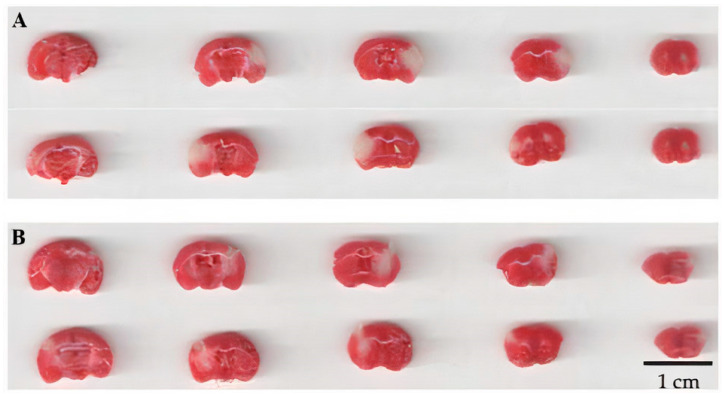
Sections of the brain of C57Bl/6 mice stained with 1% TTC. Viable tissues are colored red, while non-viable tissues remain white. (**A**) Control saline-treated animal; (**B**) animal that received KND peptide intranasally once in the first minute of reperfusion at a dose of 300 μg/kg/5 μL. The top row for (**A**,**B**) shows brain sections scanned from one side, while the bottom row shows the other side for the corresponding brain section.

Figure 6 exemplifies serial sections of the brain from representatives of the control group and the group treated with KND intranasally.

## 4. Discussion

In this study, DSIP and its synthetic analog KND peptide have been shown to possess certain protective properties against injuries resulting from occlusion-reperfusion of the left coronary artery in the rat myocardium and the right middle cerebral artery in the mouse brain. The cardioprotective effect of KND was significantly more pronounced in comparison to DSIP. For this reason, only the KND peptide was taken for further assessment of its neuroprotective properties.

One of the antioxidant mechanisms of the action of DSIP and KND may be an increase in the activity of superoxide dismutase (SOD) and glutamate peroxidase (GPO) [13], mediated by an augmentation in the expression of genes encoding these enzymes, as shown in aging animals [14].

A decrease in the volume of cerebral infarction may be due to a reduced response of the neuronal reaction to glutamate excitotoxicity. In particular, DSIP has been shown to block an increase in the neuronal activity provoked by microionophoretic application of glutamate [15].

Another study has reported the ability of DSIP to block presynaptic NMDA receptors in the culture of neuronal cells of the rat cerebral cortex, as well as to reduce glutamate- or NMDA-stimulated uptake of Ca^2+^ into synaptosomes [16]. An antihypoxic effect has been shown for DSIP in vitro, where it activated oxidative phosphorylation and increased the resistance of brain neurons to hypoxia [17].

A reduced volume of infarction in ischemic tissues may be due to the ability of DSIP (and possibly its analogs) to increase the blood flow in the tissues [4]. Increased cerebral blood flow and a decrease in mortality after cerebral ischemia were demonstrated in the study by Koplik et al. [18], in which male Wistar rats were subjected to global cerebral ischemia caused by bilateral occlusion of the carotid arteries, and preventively received DSIP at a dose of 10 μg/kg.

An intraperitoneal injection of DSIP in emotionally reactive male August rats and Wistar rats at a dose of 60 μg/kg led to a significant increase in the activity of monoamine oxidase A after 30 min in the cerebral cortex by 47% and 28%, respectively, and by 62% in both cases in the striatum, indicating activation of the serotoninergic system and inhibition of the dopaminergic system [19].

Damage of mitochondria in ischemic organs is an established fact [20,21]. For instance, ischemia-reperfusion injury of the myocardium, leading to the death of cardiomyocytes and impaired contractile function of the myocardium, includes mitochondrial damage as an important pathogenetic mechanism [22,23,24,25,26]. A decrease in respiratory activity associated with damage to complexes 1 and 111 was registered in mitochondria isolated from the ischemic brain and heart. Moreover, hypoxia inhibits adenine nucleotide translocase and ATPase [27,28]. It has also been shown that mitochondria contribute to the oxidative damage in the rabbit myocardium after ischemia [29], which leads to a reduction in the content of cardiolipin in the inner mitochondrial membranes in the sarcolemma [30,31]. A decrease in cardiolipin results in dysfunction of mitochondrial respiration through direct reduction of the cytochrome c level [30,31,32] and activates programmed cell death [33,34,35].

The mechanisms of mitochondria-mediated myocyte damage during ischemia also includes reactive oxygen species production and an increase in the permeability of mitochondrial membrane [33,36].

A number of studies have reported the therapeutic efficiency of mitochondrial transplantation in both animals [37,38,39,40,41,42,43,44] and in humans [45]. Experiments on mitochondria from the rat brain after short-term hypoxia also demonstrated a significant reduction in the rate of oxidative phosphorylation [17]. Preventive intraperitoneal injection of DSIP at a dose of 120 μg/kg completely precluded these disorders and protected mitochondria under conditions of global ischemia, modeled by placing the animals into a flow pressure chamber for 15 min under 0.26 bar, which corresponds to 10,000 m altitude above ground level [17]. The same study showed an increase in the rate of oxidative phosphorylation in the mitochondria of the rat brain homogenate, and its results suggest that the protective effects of DSIP in ischemic tissues are due to its influence on the mitochondrial ATP transport system. In our previous studies, the protective effects against cisplatin toxicity were shown for DSIP and were greater for KND [1,3]. The mechanism of the toxic action of cisplatin, which also exhibits anticancer action, was realized through oxidative stress, leading to an increase in the level of free radical oxidation and the resultant DNA damage and dysfunction of the mitochondria that in turn causes apoptosis [46]. Thus, it is logical to assume that the protective effect of DSIP and KND is aimed at blocking oxidative stress, and therefore at protecting mitochondria and their functions.

When administered intravenously in people under isoflurane anesthesia at a dose of 25 nmol/kg, DSIP reduced variability in the heart rate caused by the parasympathetic effect [47], which may also indicate one of the mechanisms of its cardioprotective action in ischemia-reperfusion of the myocardium.

The peptide under study, KND, is the 324–332 fragment (DSIP-homologous fragment) of the human lysine-specific histone demethylase 3B [1], which belongs to the JmjC-domain-containing histone demethylases that are present in the tissues of different mammals and are encoded by the *JMJD1B* gene [2]. JmjC histone lysine demethylases (KDMs) are epigenetic factors that regulate the removal of methyl groups from post-translationally modified lysyl residues within histone tails, affecting gene transcription. Oxygen is a prerequisite for the catalytic activity of these enzymes: a number of studies have confirmed that some KDMs are sensitive to the level of oxygen and can thus serve as oxygen indicators in the context of chromatin modification and contribute to the epigenetic regulation of hypoxia [48,49,50]. However, the DSIP-homologous sequence of the KND peptide originated from the 324–332 fragment, while the only catalytic fragment of JmjC is represented by the 1498–1721 sequence [1], which explains why the search for similarities in the mechanism of action of the human lysine-specific histone demethylase 3B and the KND peptide does not seem appropriate. The functional identity of the KND peptide and DSIP has already been demonstrated [3], and suggests that the mechanisms associated with the activities of DSIP are identical to those for KND.

Our data—obtained in a simulation of myocardial infarction in rats and focal stroke in mice and indicating 100% death of animals upon introduction of DSIP and KND peptides at the stage of occlusion—indicate the selectivity of the protective effect of the peptides under study depending on the type of stress factor. That is, the regulatory mechanisms triggered by the investigated peptides led to the death of the organism in acute ischemia, while exerting cardio- and neuro-protection in reperfusion. We can assume that such regulatory mechanisms include, for example, the expression of early response genes such as c-Fos. The expression of these genes takes place in response to a variety of stimuli, such as cell depolarization, exposure to neurotransmitters, growth factors, hormones, electric current, sleep deprivation, free radical oxidation, and pain among others, and the level of c-Fos expression varies between different cells and tissues. [51,52,53,54,55]. The products of these genes are transcription factors that activate the expression of a number of delayed response genes, as well as a range of cytoplasmic enzymes and secreted proteins. Subsequent cellular events are related to the processes of proliferation, differentiation, programmed cell death, and long-term potentiation, among others [51]. Data show that DSIP administered to animals exposed to immobilization stress led to a decrease in the expression of the c-Fos gene in the paraventricular hypothalamus and basolateral amygdala in rats predisposed to emotional stress [56]. The same study found this effect to be significantly less pronounced in stress-resistant animals. In other regions of the brain (the lateral septum and medial septum), the administration of DSIP led to a smaller decrease in c-Fos expression. Prevention of peptide-mediated inhibition of c-Fos expression upon administration of an NMDA receptor antagonist dizocilpine also supports the potential for DSIP to realize its effects via NMDA receptors [57].

The regulatory role of DSIP peptide in the immune system is intriguing. For example, in the myocardium of intact mice, DSIP increases the content of IL-6 by 40% without affecting the levels of IL-1 and IL-2. DSIP administration in the pituitary gland is known to cause an almost three-fold increase in IL-6 compared to intact animals, while the peptide shows no effect on the level of IL-6, but increases the content of IL-1 in the hypothalamus [58].

It has been found that under standard light conditions (12 h light/12 h dark), DSIP causes an increase in the total antioxidant activity of the brain and superoxide dismutase activity in the liver of mice. Under conditions of five-day light stress (when animals are kept under continuous illumination with no dark periods), subcutaneous administration of DSIP results in an increase in the total antioxidant activity of the liver. However, the activity of the liver antioxidant defense enzyme, glutathione peroxidase, significantly decreased under both standard lighting and stress conditions by 82% and 69%, respectively, as it was affected by DSIP [59]. The biological activities of DSIP and its structural analogs are extremely diverse and contradictory, being dependent on the stressor factor that influences the organism, as well as on its localization [60].

Overall, the synthetic structural analog of DSIP, peptide KND, is a promising drug for the treatment of ischemia-reperfusion injuries of the brain and heart, provided that it is administered during the reperfusion period and not during the occlusion period.

## Figures and Tables

**Table 1:** Myocardial infarction simulation in SD rats. Groups and doses.

Group	Drug	Dose/Injection Volume	Route of Administration	Number of Animals
1	Vehicle (saline)	5 mL/kg	intraperitoneally	5
2	KND	150 μg/kg/5 mL	intraperitoneally	8
3	DSIP	150 μg/kg/5 mL	intraperitoneally	8

**Table 2:** Experimental groups and respective doses for C57Bl/6 mice in the stroke simulation.

Group	Drug	Dose/Injection Volume	Route of Administration	Number of Animals
1	Vehicle (saline)	5 mL/kg	intraperitoneally	8
		5 μL/mouse	intranasally	8
2	KND	300 μg/kg/5 mL	intraperitoneally	12
3	DS KND IP	300 μg/kg in 5 μL/mouse	intranasally	8

## Data Availability

All raw data are presented in Appendix A.

## Appendix A

### Appendix A.1. Raw Data on Heart Infarction

**Table A1:** Saline 0 Min of Reperfusion.

Animal Number	Area at Risk (pxl)	Infarct Size (pxl)	IS/AAR (%)	AAR/LV (%)
1	183,178	96,205	52.5	39.7
2	197,700	96,841	49.0	41.9
3	147,014	44,366	30.2	38.4
4	185,200	78,850	42.6	35.1
5	120,950	43,561	36.0	33.0
Mean ± SD	166,808 ± 31,841	71,965 ± 26,563	42.1 ±9.2	37.6 ± 3.6

**Table A2:** DSIP 0 Min of Reperfusion.

Animal Number	Area at Risk (pxl)	Infarct Size (pxl)	IS/AAR (%)	AAR/LV (%)
6	131,333	29,030	22.1	38.7
7	141,290	20,690	14.6	50.7
8	125,025	54,818	43.8	16.4
9	157,725	43,169	27.4	36.19
10	168,022	60,736	36.1	42.46
11	179,456	63,629	35.5	30.84
12	144,298	33,708	23.4	32.4
13	190,906	50,938	26.7	44.6
Mean ± SD	154,757 ± 17,965	44,590 ± 16,871	28.7 ± 11.5	36.5 ± 17.4

**Table A3:** KND 0 min of reperfusion.

Animal Number	Area at Risk, pxl	Infarct Size, (pxl)	IS/AAR, %	AAR/LV, %
14	160,999	45,037	28.0	37.9
15	145,299	25,736	17.7	29.0
16	172,489	40,656	23.6	39.9
17	219,653	46,644	21.2	44.5
18	195,348	51,709	26.5	43.7
19	142,787	10,276	7.2	39.9
20	134,033	22,997	17.2	30.6
21	128,607	14,278	11.1	32.2
Mean ± SD	162.402 ± 29.248	32.167 ± 9.892	19.1 ± 4.1	37.2 ± 6.2

### Appendix A.2. Raw Data on Cerebral Stroke

**Table A4:** Middle cerebral artery occlusion after 24 h reperfusion and saline.

Animal Number	Right Hemisphere Volume (mm^3^)	Right Hemisphere Damage Volume (mm^3^)	Right Hemisphere Damage Volume (%)
1	0.229	0.022	9.657
2	0.220	0.012	5.495
3	0.227	0.034	14.832
4	0.206	0.023	11.392
5	0.214	0.033	15.218
6	0.192	0.045	23.660
7	0.190	0.019	9.966
8	0.215	0.053	24.688
9	0.207	0.029	14.130
10	0.211	0.016	7.704
11	0.209	0.012	5.955
12	0.206	0.020	9.544
13	0.209	0.023	10.851
14	0.206	0.024	11.635
15	0.216	0.014	6.290
16	0.218	0.029	13.456
Mean ± SD	0.211 ± 0.011	0.026 ± 0.011	12.155 ± 5.592

**Table A5:** Middle cerebral artery occlusion after 24 h reperfusion and KND 300 µg/kg administered intraperitoneally.

Animal Number	Right Hemisphere Volume, (mm^3^)	Right Hemisphere Damage Volume, (mm^3^)	Right Hemisphere Damage Volume (%)
17	0.203	0.001	0.604
18	0.211	0.011	5.195
19	0.216	0.014	6.555
20	0.207	0.021	10.393
21	0.214	0.027	12.603
22	0.196	0.013	6.437
23	0.205	0.024	11.816
24	0.200	0.040	19.801
25	0.218	0.030	13.628
26	0.211	0.022	10.505
27	0.208	0.013	6.325
28	0.207	0.013	6.056
Mean ± SD	0.208 ± 0.007	0.019 ± 0.010	9.160 ± 5.011

**Table A6:** Middle cerebral artery occlusion after 24 h reperfusion and KND 300 µg/kg administered intranasally.

Animal Number	Right Hemisphere Volume (mm^3^)	Right Nemisphere Damage Volume (mm^3^)	Right Hmisphere Damage Volume (%)
29	0.209	0.011	5.250
30	0.224	0.014	6.180
31	0.213	0.019	9.144
32	0.218	0.023	10.762
33	0.221	0.014	6.136
34	0.206	0.028	13.427
35	0.220	0.012	5.477
36	0.210	0.005	2.519
Mean ± SD	0.215 ± 0.007	0.016 ± 0.007	7.362 ± 3.503
